# A Practical Treatise on Diseases of the Uterus and Its Appendages

**Published:** 1834-10-01

**Authors:** 


*A Practical Treatise on the Diseases of the Uterus and its Appendages. Translated from the French of* M$^{me}$ VEUVE BOIVIN *and* A. DUGÈS: *with copious Notes, by* G. O. HEMING, F.L.S., Consulting Accoucheur to the St. Pancras Infirmary, &c. —London, 1834. 8vo. pp. 559; and a volume of Plates.

THE morbid anatomy of the unimpregnated uterus has of late years attracted much attention, and has consequently received great improvements; but it is to be regretted that our diagnosis of these diseases during life has not made a corresponding progress. This may indeed be partially attributed to the fashion which, since the time of Dr. Baillie, has pervaded the profession, of studying morbid anatomy to the exclusion of other equally important parts of the science of medicine. But, unless we are greatly mistaken, the numerous errors which are daily committed in treating uterine diseases do not arise from causes of such a general nature, but rather from circumstances peculiar to the affections of this organ. We pass over the number of these diseases and their intrinsic complexity, as any observations on these topics would rather tend to paralyse than to stimulate exertion. We pass over the neglect of education on these points in our hospitals and dispensaries, as we do not flatter ourselves with possessing sufficient influence to alter the routine of such institutions, — we pass over these, which all have their influence in obstructing our knowledge of these affections, to dwell upon other circumstances which admit of remedy, and from the consideration of which we may therefore hope to derive some beneficial results.

In the first place, though their pathology has been much studied, yet we are not in possession of any scientific classification of diseases of the uterus; that is to say, one founded on the morbid changes of its structure. We do not wish in any manner to derogate from the utility or excellence of Sir Charles Clarke's work; on the contrary, Mr. Heming has expressed our own sentiments, in his preface, where he says, "The work of Sir Charles Clarke has appeared to me of the greater value the more I have perused its pages. It is, as is well known, chiefly confined to the inflammatory or organic diseases of the uterus and its appendages, and is the best which can be consulted upon this part of the subject." To him we owe the removal of leucorrhœa and menorrhagia from the catalogue of diseases, and their enrolment in that of symptoms, to which they properly belong; yet it seems to us, that he retains some of the old leaven, when he makes these discharges the basis of his classification. In fact, it often happens that our modern systems are old phantasies under a more seemly mask; just as we are sometimes told that the Church of Rome has retained the worship as well as the gorgeous ceremonies of its pagan predecessors, and that Saint Somebody is merely a modern name for Pan or Vesta. We are willing to confess that there is some advantage in his arrangements, as discharges do admit of a careful examination by the practitioner, and may therefore assist him in guessing at the nature of the complaint; but there are some, even organic diseases, which in their onset are not accompanied by discharge of any kind, and there are others which, in their various stages, are attended by discharges of very dissimilar characters. It may be said that, in attributing an imperfect diagnosis to the want of a scientific classification, we are laying too much stress upon such arrangements, and that nosology is totally distinct from the practice of physic. To this we reply, that a classification founded upon the pathological characters of disease does not stand on the same footing with ordinary nosologies. They are the only firm foundation from which investigations may safely start, while at the same time they assist in giving correct notions of the nature and treatment of disease. Who will deny that surgeons know more of dislocations and their diagnosis since their excellent classification by Sir Astley Cooper? or who will venture to affirm, that our knowledge of diseases of the joints has not been greatly improved by the scientific arrangement of Mr. Brodie. Neither would such a classification be attended with more difficulty in diseases of the uterus than in those of any other organ, excepting inasmuch as the former are modified by the pregnant and puerperal states. These, however, would rather add to the labour than increase the difficulty; the field would not be more stubborn, though it might be more extensive. On the contrary, could we persuade some competent person to undertake the task, we believe that he would not find the labour excessive, as vast quantities of excellent materials have been already prepared by many distinguished pathologists. Let us, however, not be misunderstood: we do not recommend any young physician or surgeon, whose time may hang heavily on his hands, to commence collating and extracting from the works of other writers, according to the established method of book-making. The attempt could only be brought to a successful issue by one who has studied those diseases at the bedside, and, having acquired by experience the power of separating the wheat from the chaff in the writings of others, has also accumulated sufficient materials of his own to give an original stamp to his productions. Without such a classification some few practitioners, by very extensive experience, may acquire a knack of detecting most of these diseases, and they may conduct an empirical mode of treatment with tolerable success; but their less fortunate brethren, without the same opportunities, and without any scientific basis on which to form their opinions, are and must be continually at sea; hence arise the ignorance and confusion which prevail upon the subject, and hence also the maltreatment of most of these complaints.

There is another circumstance which is adverse to our progress in distinguishing these diseases, but which ought indeed to have a directly opposite tendency; we refer to the power of examination by the touch. "The eye of the practitioner should be situated in the extremity of the forefinger of the right hand," was the pithy expression of a late celebrated lecturer on the diseases of women; and, at the period at which such language was employed, it conveyed excellent advice to practitioners who utterly neglected this means of diagnosis. The knowledge thus acquired has been found so valuable, that the study of the other symptoms not cognizable by manual examination has been nearly abandoned.

A few general terms applicable to all the diseases of this organ are still retained, such as weight about the hypogastrium, pains in the loins, &c.; but these are so vaguely and indiscriminately applied, that no value can be attributed to them in practice; and, should a surgeon, in consultation, presume to throw out a surmise as to the nature of the complaint from an accurate detail of the general symptoms, it would certainly be disregarded, if it did not expose him to the derision of his fellow practitioners. Yet we do not hesitate to affirm that such a surmise may be made with tolerable accuracy: we would even go further, and say that some general opinion as to the nature of the disease should be formed prior to the examination by the finger, not only for the purpose of shortening a procedure so disagreeable to the patient, but also to assist in arriving at a correct conclusion.

It is not the study of these diseases alone that has been injured by this mechanical system of physic, though perhaps it has suffered most from its effects. Since the application of the stethoscope to the diagnosis of diseases of the chest, their general symptoms have been too much overlooked; indeed, we ourselves remember to have seen a physician of eminence in Paris, while making his daily visit to the hospital, perambulate the wards with the stethoscope in his right hand, and a yard measure in his left, as if in these two instruments he carried all that was necessary for the practice of physic. In England, however, the sound sense of our countrymen (not to speak of the *amour-propre* of those who possess an exquisite tact of diagnosis by other means,) has partially arrested this mania for listening and measuring, and prevented the utter disregard of that knowledge which has been accumulated by the experience of ages. No one would advocate the discarding of the stethoscope in diseases of the chest, or the manual examination in those of the womb; but we would remark, that medicine is a cumulative science, that every fresh means of diagnosis is valuable as an addition to our stock, but that its value is greatly diminished if its adoption is to be followed by the neglect of others formerly in use, and of whose utility we have by long experience been convinced.

Another mechanical aid has of late been much in vogue, viz. the *speculum uteri*, but we believe that its utility has been greatly overrated. We are willing to concede that, where ulceration of the os uteri exists, this instrument, by enabling us to examine the surface, may greatly contribute to prove our diagnosis; but it can be of scarcely any service in distinguishing the various tumours to which these parts are liable before the period of ulceration. Meanwhile it is open to severalo bjections. Very few patients will submit to its employment, and others are frequently put to considerable pain by its use. Besides, we have seen bleeding fissures of the os uteri, which we have been strongly inclined to attribute to the speculum; and we conceive that tumours having a tendency to ulcerate, would not have that tendency diminished by the lancinating powers of this instrument.

Having thus stated what we deem to be the bars to our improvement in these diseases, let us examine how far the work before us is calculated to supply the desiderata.

The preface, by Mr. Heming, contains a slight sketch of the recent improvements in this department of medical science; which is followed by an introduction, from the pens of Madame Boivin and Professor Dugès, in which the structure of the healthy uterus, and its appendages, is considered in the infantile, virgin, pregnant, and puerperal states. The work itself is divided into two parts, and the first is devoted to diseases of the uterus; these are subdivided into, breaches of continuity, changes of situation, deviations in form and volume, distension of the uterus by foreign bodies, excrescences and morbid changes of structure, acute and chronic inflammation of the uterus, irregularity of the catamenia, and uterine neuroses. The second part, which comprises the diseases of the appendages, treats separately of those of the ovaria, of the Fallopian tubes, the vagina, and the pudenda. This classification is a first step to improvement, as it is partially based upon the changes of structure; nevertheless, it contains many obvious errors. For instance, the inflammation of the womb, with which the work should commence, in order that the reader may be familiar with its symptoms, as it is developed in the course of other diseases, does not find a place till near the end of the volume. But there are others which we deem more important: for instance, prolapsus uteri is but a symptom of primary extension of the ligaments of the womb, or of relaxation of the vagina and neighbouring parts. We would therefore have these affections distinguished, in order that the practitioner may recognize the curable from the incurable cases. We must, however, pass on to our review of the work itself; but as, from its great extent, and the number of its references and quotations, it rather deserves the name of a Cyclopædia of Diseases of the Uterus than that of a practical treatise, we shall not pretend to follow the authors throughout the whole of their work, but, having selected a few passages by which our readers may judge of its style and execution, we shall make a few comments on the doctrines which it puts forth.

At the very outset of the book, in the introduction, we meet with a fault which demands the severest reprehension. In the section on the *Uterus considered externally*, the following passage occurs: "When she is in the supine position, [the womb] does not extend beyond the level of the brim of the pelvis, and cannot be felt through the abdominal parietes, excepting when these are extremely thin and lax; the top of the finger then reaches it, and experiences the resistance, sometimes very obscure, of a body of round form, moveable, and yielding to pressure, and pretty hard, the volume of which it is therefore difficult to determine, yet such as not to admit of being felt by more than two fingers at once." (P. 3.) "The latter part of this description," says the translator, "appears to me a little imaginative." We cannot, however, dismiss the passage with this mitigated censure. The uterus, in its healthy unimpregnated state, cannot be felt through the parietes of the abdomen; and the authors, in this instance, as in several others, have evidently written not from their experience, but from their fancy, and stated what should be, instead of what is. We have said that the above extract is not a solitary instance of this iniquitous practice. At page 46, while detailing the symptoms of *Prolapsus uteri*, the following passage occurs: "The somewhat empty space left in the pelvis by the prolapsus uteri may at the same time be detected by the application of the hand to the hypogastrium; and this will be the best means of distinguishing prolapsus from elongation of the cervix."* We do not hesitate to say, that there is not one case out of a hundred in which any empty space can be felt through the abdominal parietes, and that the authors have coined the fact in order to make up their tale. This is a fault too common in medical writings, and cannot be too harshly reprobated. It invalidates the author's testimony on other points, and strikes at the very root of inductive philosophy, by diminishing our credence in narrations of facts.

By way of preparation for the study of the diseases, the authors give a good description of their method of examining the uterus, which we quote, with the additional observations of the translator.

"1. *Super-pubic Examination.* The patient being conveniently placed in the supine posture, the head and the shoulders a little raised, and the thighs semi-flexed upon the abdomen, the hypogastrium should be carefully pressed in every direction by the hand. If a hard body be felt, the fingers should be applied so as to ascertain, if possible, its volume, form, consistency, mobility, and connexion with other organs. The iliac fossæ should be first examined, where the Fallopian tubes and ovaria are sometimes found, when diseased: afterwards, the hypogastrium, which frequently contains the fundus uteri. It will be necessary, in some cases, to press the abdominal parietes deeply into the pelvis with the bended fingers, while the palm of the hand is applied to the fore part of the pubes. The bladder and the large intestines should always be previously evacuated.

"2. *Examination per Vaginam.* The erect posture is the most convenient for ascertaining the weight, elevation, and direction of the uterus: in other respects, the supine posture is better, as in the former examination.† The forefinger is generally sufficient for

* It is but justice to Mr. Heming to quote his note upon this passage, as it exemplifies the practical nature of all his observations: "Elongation of the cervix uteri is best ascertained by tracing it with the finger introduced first in vaginam, and then into the rectum; or, if pregnancy does not exist, by passing a probe into the os uteri."

["† It is usual to make the examination per vaginam, as in labour, the patient being on her left side. But I am persuaded that there are, in many cases, great advantages in the position upon the back, the head and shoulders being raised: 1, in this manner the uterus falls a little lower; 2, the sentient part of the finger receives the os uteri and especially its posterior border, and the anterior part of the vagina, whilst it is easily passed to each side; and 3, the hand is easily revolved, so that the finger may touch the anterior part of the cervix and the posterior paries of the vagina. This is peremptory when we want to obtain repercussion, and to examine prolapsus, in which the patient must also be raised almost into the erect position."—Tr.]

this examination, whilst the middle finger, bent towards the palm of the hand, rests upon the perinæum and presses it upward. In some cases the whole hand must be introduced into the vagina. The finger is passed along the canal, under the cervix uteri and behind it, pressing the several parts, to ascertain their sensibility; it then raises the whole uterus, to form an estimate of its weight and mobility, and then ascertain its size, the other hand being pressed upon the hypogastrium. On withdrawing the finger, a white napkin is used, in order to ascertain the state of the discharges. The rectum should be previously evacuated; as it otherwise flattens the vagina, forces its posterior paries forward, and renders it difficult to reach the body of the uterus. This will be best done by an abundant enema of warm water.

"3. *Examination per Rectum.* This mode of examination is adopted only when the preceding one furnishes an incomplete diagnosis. Cases will occur in which tumors, formed between the uterus and the intestine, or towards the lateral and posterior parietes of the pelvis, congestions of the body of the uterus, and particular displacements of this organ, cannot be sufficiently examined per vaginam. Cases also of imperforation, and excessive narrowness of the vaginal orifice, may require examination per rectum; and we have already shewn its usefulness in cases in which the uterus was entirely wanting.

"The patient should be placed in the same position as in the preceding examination, with the pelvis raised. The forefinger should be introduced into the rectum." (P. 31.)

A short and homely injunction of the late Dr. J. Clarke to his pupils is worthy of being added to the above directions, as it contains a caution against the most common error of young practitioners. "Never withdraw your finger till you have completed your examination, and satisfied yourself upon every point which you wish to ascertain."

Among the causes of *Procedentia Uteri,* which are extremely well detailed, our authors mention the sudden displacement of the uterus by muscular exertion, violent pressure on the abdomen, the shock in jumping, or a fall upon the nates, &c. This sudden displacement is a fact that has been generally overlooked, but to the truth of which we can give our testimony. It occurred in a young woman after violent straining, but in her case was followed by metritis of the most intense character. The liability to inflammation after accidents of this kind should have been stated in the work.

The subject of displacement of the uterus occupies nearly a fourth of the volume, and has evidently been the product of much labour and attention. We shall abridge the description of *Anteversion of the Uterus,* as it is not only the best which we possess, but also one of the best parts of the treatise.

The anteversion of the uterus is frequently overlooked by writers and practitioners, on account of their attention being exclusively occupied by the disease with which it is generally complicated. The slight inclination of the uterus forwards when the bladder is empty is neither inconvenient nor morbid, but, when the weight of the fundus and anterior part is increased by congestion, the utero-iliac folds of the peritoneum are gradually extended, and the uterus itself falls forward, its upper part pressing on the bladder, and its cervix on the rectum. It rarely happens in pregnancy, as the womb is early carried above the pubis, and then, if it incline forwards, it constitutes what is commonly termed *obliquity*. One case, however, of anteversion of the pregnant uterus is recorded by Baudelocque; and M. Boivin has met with another in the second month of pregnancy, but the reduction took place naturally by the progress of gestation. After parturition, a condition between obliquity and anteversion occasionally occurs from violent efforts, or premature exercise on foot. Again, anteversion may be produced without any congestion, by repeated straining or vomiting, or by difficult defæcation, or a loaded state of the rectum; or, lastly, by morbid attachments, the consequence of metritis and peritoneal inflammation. The following is the authors' account of the symptoms which it produces:

Pain is "generally felt in the lumbar and epigastric regions, owing to the dragging of the ovarian plexuses, which proceed from the great sympathetic. The patient is conscious of a weight, when walking or sitting, about the rectum and bladder: the os uteri presses on the intestine, the fundus rests upon the bladder, and forces it to evacuate itself more frequently than usual; sometimes it even falls lower, and presses upon the neck of the bladder, inducing retention of urine (*Desgranges*); while the os uteri, forced backward, sometimes arrests the passage of the fæces along the rectum. These inconveniences, which cease, for the most part, when the patient lies upon the back, unless there be adhesions of the uterus, have often masked the nature of the disease. Stone in the bladder has been suspected; and it was not until Levret had performed an unsuccessful operation of lithotomy, that he verified, for the first time, the existence of anteversion: the tumor, formed in the bladder by the fundus uteri, is not, however, so hard as a stone, nor does it give the sensation of a calculus, on passing the sound; and, on examination per vaginam, the finger feels the anterior surface of the uterus, when the patient stands up or sits on the edge of a chair: sometimes the vagina is partly obstructed by the fundus uteri inclining forward, either directly, or a little more on one side than the other, so as to constitute a sort of latero-version, the only one of that kind, perhaps, which can exist. The os uteri is always very backward, and must be sought in the concavity of the sacrum: though generally fallen with the rest of the uterus, it is sometimes raised so high as to be hardly reached by the finger. If the os uteri be brought forward by the top of the bended forefinger, and the fundus pushed back by it, it is easy to restore the organ to its natural direction, though it immediately falls back into its unnatural position. In one case only, that of adhesions caused by inflammation, this replacement cannot be so quickly effected; but, independently of this, the pelvis presents no obstacle to reduction: the pubes, inclining forward and downward, allow the fundus uteri, even when swollen, to pass easily, an advantage not so favorably afforded by the sacrum in cases of retroversion. In these circumstances, we may generally ascertain that the uterus has more weight, volume, and sensibility than usual. This state of congestion or of chronic inflammation, which we consider, with Levret, a frequent cause of anteversion, may, sometimes, be the actual effect; and this has appeared the more common event to several practitioners, and, among them, to Désormeaux, according to whom, chronic metritis frequently appears only when the disease has been of rather long continuance: it is equally certain that acute metritis, after labour or a fall, may pass into a chronic state and produce anteversion, which will only yield with the original disease." (P. 65.)

This inflammatory action is inimical to the use of the pessary, though it is not so invincible an objection as might have been supposed. The application of leeches to the pudenda, emollient fomentations and baths, narcotics, and rest in the horizontal position, with the pelvis slightly raised, will generally put a stop to the metritis where the pessary is used, and, where it is not, will frequently succeed in curing the disease. The method of employing the pessary in these cases is thus described:

"The pessary generally used, in cases of anteversion, is the cup-and-ball, having a deep cavity to receive the cervix uteri. The proper position of the uterus will be restored by pushing up the fundus and drawing down the cervix, either with the finger or with the *cuiller fenêtrée* (windowed spoon): it is preserved by keeping the patient on her back, and by pressing with one hand upon the hypogastric region, as deeply as possible, whilst the pessary is introduced with the other; the cervix is made to enter into the cup, by repeated movements from before to behind, while the finger ascertains its position: being finally adjusted, the instrument is still further introduced, and placed in the axis of the vagina. The *bung-shaped* pessary, especially the *élytroïdes* of M. J. Cloquet, may suffice for the treatment of less important cases; in others, more rare, a small sponge passed into the vagina, behind the os uteri, when very prominent, has served to support the uterus when slightly displaced." (P. 67.)

The use of the pessary should be continued till the leucorrhœa occasioned by it ceases, which is generally a year or fifteen months. Pregnancy may sometimes, though it does not always, prove a cure.

Several cases are then related, which establish the truth of the above description; and a very good representation of this displacement is given in the fifth figure of the eleventh plate.

From the section on Changes of Structure in the Uterus, we shall select for examination the chapter on *Tuberous Cancer*, or *Cancerous Tumours*.

The authors complain, with justice as regards continental surgery, that the term cancer is used to express morbid structures of totally different natures; but it must be confessed that they take a most extraordinary method of remedying the evil, viz. to discard altogether, in their employment of the term, any idea of a distinct anatomical texture. The translator, too, imbued (as translators should be) with a double portion of the author's spirit, has taken care that his language, by its obscurity and indistinctness, should be in strict accordance with the vagueness and confusion of the definition. We shall leave to our readers the task of unriddling the meaning of the passage so ingeniously complex.

"G. *Cancer*. This term has been expunged from the vocabulary of pathological anatomy, owing to the want of precision in its use: we shall therefore prefer using it as a means of embodying some purely practical remarks, separate from any anatomical data, which would be vague, incomplete, and very inapplicable to clinical observations. By *cancerous* we shall designate every affection which, by converting, in its progress, the texture of the uterus, has a natural tendency to increase, to propagate itself all around, and ultimately to destroy itself by ulceration beginning at its centre." (P. 176.)

The authors then proceed to divide cancer into the scirrhous, the fungous, the ulcerous, and the hæmatode. We should not object to this nomenclature more than to that now in use, if it were not that, by admitting these as the only forms of malignant disease of the uterus, we are rather receding than progressing in our knowledge. Dr. Baillie has described briefly, but most accurately, a tumour of the uterus which does not terminate in ulceration, to which he has given the name (whether properly or improperly we shall not stay to determine,) of scirrhus. He has marked out its symptoms so clearly, that it might be recognized with facility during the life of the patient. The authors, however, in treating of tuberous cancer, confound this disease with the earlier stages of the ulcerating cancer of the womb, though their symptoms and subsequent course are totally different. Again, they make no distinction between the ulcerated carcinoma and the corroding ulcer of Clarke: nevertheless, these diseases are essentially distinct in their pathology and symptoms. We shall lay no stress upon the union, under the head of fungous cancer, of the cauliflower excrescence and the fungous hæmatodes, as it is still a mooted point whether these be not one and the same disease, and the authors have a right to adopt whichever side of the question they may think proper; but we deem it our duty to protest against the voluntary resignation of knowledge which has cost so much labour to acquire, because, forsooth, there is yet something which remains unknown. The causes of tuberous cancer are thus detailed.

"The uterus, naturally liable to periodical congestions, to sudden and considerable changes in nutrition and vitality, must, in consequence, be much more exposed than many other organs to these diseases of texture. Of 707 cases of cancer observed in the principal organs of the body, those of the uterus constitute more than half, amounting in fact to 409. The organs of generation are the principal seat, in both sexes, of this alarming disease: in the female it usually happens most frequently in the uterus, then in the ovarium, and then in the mamma.

"The development of cancer in the uterus is found to be analogous, in frequency of occurrence, with the degree of activity of that organ: thus, the disease appears invariably after the period of puberty, and especially towards the term when its functions are about to cease, seldom showing itself when the uterus has become inactive from old age. The periods of its most frequent occurrence are first, from the fortieth to the fiftieth year, then from the twentieth to the thirtieth, and, lastly, from the thirtieth to the fortieth; and it has been less frequently observed in early youth than at the periods quoted, though much more so than after the fortieth year.* Persons who are pale, thin, nervous, and phlegmatic, labouring under tuberculous disease, subject to periodical depression, and of

* "In the 409 cases of cancer of the uterus, we have made the following enumeration:

| | |
|---|---|
| Under twenty years of age | 12 |
| From twenty to thirty | 83 |
| From thirty to forty | 102 |
| From forty to forty-five | 106 |
| From forty-five to fifty | 95 |
| From fifty to sixty | 7 |
| From sixty to seventy-one | 4 |
| Total | 409 |

solitary inactive habits, are more subject than others to cancer of the uterus. Though found to occur in the unmarried, it is of much rarer occurrence in such persons than fibrous diseases, which it resembles in its great induration and slow progress. It is a fact that local violence tends to produce this disease. It has not been observed that repeated labours constitute a particularly frequent cause, though the contrary remark has been made in the case of abortions, irregularity of the catamenia, and repeated menorrhagia, either because these affections are the cause, or the effect, of congestion of the uterus, or of scirrhus of this organ.* It is very evident, in fact, that whatever tends to maintain the uterus in a state of such congestion, or of chronic inflammation, may at length give rise to cancer: syphilis has been known to lead to it indirectly; but we are speaking now rather of the ulcerated than of the tuberous form.

"Cancer very often appears without any assignable cause. MM. Bayle and Cayol observe, "We have known persons of the greatest profligacy to die of cancer of the uterus,—unmarried women, in cases in which the hymen was perfect,—married women who have borne many children, and others who had never been pregnant." This disease is sometimes the result of a diathesis already manifested by scirrhus of the mamma; sometimes, without affording any distinct signs, it appears to proceed from hereditary constitution." (P. 231.)

There is a strange misstatement in this passage. The text informs us that the period from twenty to thirty years of age is more liable to this disease than any, excepting that from forty to fifty: the note, on the other hand, asserts it to be more rare from twenty to thirty than from thirty to forty. As far as our own experience goes, we are inclined to believe the note, which, we think, rather exaggerates than diminishes the proportion of patients affected in early life.

The account of the course and symptoms of tuberous cancer is necessarily very imperfect, as there is no attempt at discriminating each form of the disease. One observation is however very good, and deserves quotation.

"Hæmorrhagy is a most important symptom, when observed in a person who has exceeded the period of the catamenia; and some cases which we have seen prove the necessity of rigorously exa-

"* In all cases where frequent abortion or premature labour takes place, the uterus should be examined: it will very frequently be found to be diseased.

"This may be expected to be the case, more especially if there be frequent, very slight discharges of blood, during the early months of pregnancy. In two cases of this kind, which recently occurred to me, I was induced to examine the uterus, in consequence of the discharge continuing some weeks after the abortion had taken place: in both cases I found disease. In the first, premature labour took place three times with dead children: the other case resembled it in many points, but in this the ovum was expelled four times at the fourth month. The disease was carcinomatous, and ultimately destroyed the patients.—TR.

mining into those pretended renewals of the catamenia spoken of by physiologists. This apparent return of youth is in general only a sign of some important disease in the uterus or its appendages; it has often been observed to be followed by a rapid wasting and sudden death. There are also a great many persons who are continually subject to augmentations and diminutions (sometimes even periodical,) of abundant aqueous discharges, inodorous or of a faint smell; slightly charged with albumen, as is seen by the slight stiffness, the somewhat greyish colour which it imparts to the linen on drying, and of a slight rose colour at the period of the catamenia. This serous and profuse discharge appears to us to announce, if not the existence, at least the commencement or the approach of ulceration, of fungous growths, constituting the third degree or period of this disease." (P. 234.)

There is but little novelty in the palliative treatment recommended by the authors, but their observations on the curative treatment are copious, and so marvellous, as to excite even the translator's admiration. The various methods of procedure are cauterization, and partial and total excision. The first is to be employed only in incipient cases, as otherwise they have an unfortunate tendency to lay the vagina, bladder, and rectum into one cavity, not to mention the liability to peritonitis, or the danger of hemorrhage. The partial excision is fully discussed according to the methods of Dupuytren, Lisfranc, Mayor, &c. This operation is somewhat discountenanced by the authors, as only one case in six survives its performance; nevertheless, they would occasionally permit it "in cases in which the cancer is insupportably painful." We leave our readers to judge how nearly this permission approaches to authorizing practitioners to decide whether the patients' lives are worth preserving, and to put a speedy end to them, provided that the decision is in the negative. But this is not all the humanity of the authors: "*in the hope of affording occasional relief*," they actually proceed to add a few words "on the superpubic and subpubic extirpation of the whole uterus." The only cases on record of the former operation died within a few hours, and the success of the latter appears to be no greater.

Mr. Heming's note, at the conclusion of the chapter, is creditable both to his judgment and feelings. "These difficulties, together with a real blot upon surgery, would be best removed by entirely confining these barbarous operations in future to the dead subject." (P. 253.)

We must now close our notice of the work. Mr. Heming has added many excellent observations, both from his own experience and from the writings of the best authors of this country, and, whenever he has stated his opinions, they appear to be sensible and to the purpose. We have, however, two small objections to make: first, that we occasionally meet with expressions which partake quite as much of the French as of the English language, having the words of the one and the idiom of the other; and, secondly, that the omission of several passages, on the ground of their want of chastity, looks much like a quiz on the modesty of medical men, while it forms a grievous libel on the purity of the lady-author.

The lithographic plates are of great assistance in understanding the authors' descriptions; they are exceedingly cheap, and very fairly executed.

